# Russeting in Apple Is Initiated After Exposure to Moisture Ends—I. Histological Evidence

**DOI:** 10.3390/plants9101293

**Published:** 2020-09-30

**Authors:** Yun-Hao Chen, Jannis Straube, Bishnu P. Khanal, Moritz Knoche, Thomas Debener

**Affiliations:** 1 Institute of Horticultural Production Systems, Fruit Science Section, Leibniz University Hannover, Herrenhäuser Straße 2, 30419 Hannover, Germany; chen@obst.uni-hannover.de (Y.-H.C.); moritz.knoche@obst.uni-hannover.de (M.K.); 2 Institute of Plant Genetics, Molecular Plant Breeding Section, Leibniz University Hannover, Herrenhäuser Straße 2, 30419 Hannover, Germany; straube@genetik.uni-hannover.de (J.S.); debener@genetik.uni-hannover.de (T.D.)

**Keywords:** russeting, periderm, *Malus* × *domestica*, surface moisture, cuticle, strain

## Abstract

Russeting (periderm formation) is a critical fruit-surface disorder in apple (*Malus* × *domestica* Borkh.). The first symptom of insipient russeting is cuticular microcracking. Humid and rainy weather increases russeting. The aim was to determine the ontogeny of moisture-induced russeting in ‘Pinova’ apple. We recorded the effects of duration of exposure to water and the stage of fruit development at exposure on microcracking, periderm formation and cuticle deposition. Early on (21 or 31 days after full bloom; DAFB) short periods (2 to 12 d) of moisture exposure induced cuticular microcracking—but not later on (66 or 93 DAFB). A periderm was not formed during moisture exposure but 4 d after exposure ended. A periderm was formed in the hypodermis beneath a microcrack. Russeting frequency and severity were low for up to 4 d of moisture exposure but increased after 6 d. Cuticle thickness was not affected by moisture for up to 8 d but decreased for longer exposures. Cuticular ridge thickness decreased around a microcrack. In general, moisture did not affect cuticular strain release. We conclude that a hypodermal periderm forms after termination of moisture exposure and after microcrack formation. Reduced cuticle deposition may cause moisture-induced microcracking and, thus, russeting.

## 1. Introduction

Russeting is a commercially important surface disorder of many fruit crop species, worldwide. Among other species affected are: apple [1], pear [2], grape [3] and prune [4]. The rough, brownish appearance of russeting renders a fruit unattractive to the consumer. Russeting also increases rates of postharvest moisture loss that lead to shriveling (fruit lose their fresh glossiness, so look old) and to higher rates of mass loss during storage, transport and retail (fruit are priced to the consumer on a per-kg basis) [5].

In anatomical terms, russeting represents a periderm comprising the phellem, a phellogen and a phelloderm [6,7]. The phellem cells (also referred to as cork cells) have suberized cell walls that are responsible for the dull and brownish color of a russeted fruit. These cork cells typically occur in stacks, resulting from division of the phellogen cells [8]. 

Information on how such a periderm is initiated in apple fruit skin is limited. Empirical evidence indicates that a range of factors may be involved. These include mechanical wounding [9], certain agrochemicals [10,11,12], epiphytic microorganism [13], insects (rust mites) [14] and diseases [15]. Of particular interest here is the effect of moisture on russeting in apple. Numerous studies indicate that exposure to surface wetness [16,17,18] or to high humidities [19] can be the cause of russeting in apple. Surface moisture, applied either as liquid-phase water or as vapor-phase water, induces microcracking in a number of fruit crop species, including apple [16]. Microcracks in the apple fruit skin are the first visible symptom of insipient russeting [20,21,22]. The mechanism of water-induced microcracking is not clear. It is possible that one of the factors is modification of the mechanical properties of the cuticle induced through changes in hydration [23]. 

We recently developed a system that reliably induces microcracking and russeting by local exposure of patches of the apple fruit surface to moisture [24]. Briefly, a length of tube is attached to the fruit surface using a non-phytotoxic silicone rubber. The tube is filled with water and periodically resealed to the fruit surface. The patch of skin included within the tube footprint first develops microcracks and, later, displays symptoms of russeting. These symptoms are microscopically identical to those observed on a fruit naturally exposed to surface moisture in the field. This system may be helpful in studying the mechanistic basis of russeting. It also avoids confusions associated with comparisons of different fruit genotypes or of different individual fruit or of different regions on the fruit surface. It allows critical comparisons to be made by imposing a moisture treatment to a defined patch of fruit skin, while an untreated (control) patch is defined in an equivalent region on the surface of the same fruit. It thereby allows standardization for a range of potential sources of response variability including stage of fruit development, differences in micro-environment, in orientation and in management (tree center vs. periphery etc.).

The specific objectives here were to identify the sequence of events that culminate in moisture-induced russeting. We were particularly interested to determine when and where a periderm is formed in relation to the location of moisture exposure. We focused on apple because apples are an important fruit crop species in both the northern and southern hemispheres and because russeting presents a problem to producers of this fruit crop. 

## 2. Results

**Figure 1:**
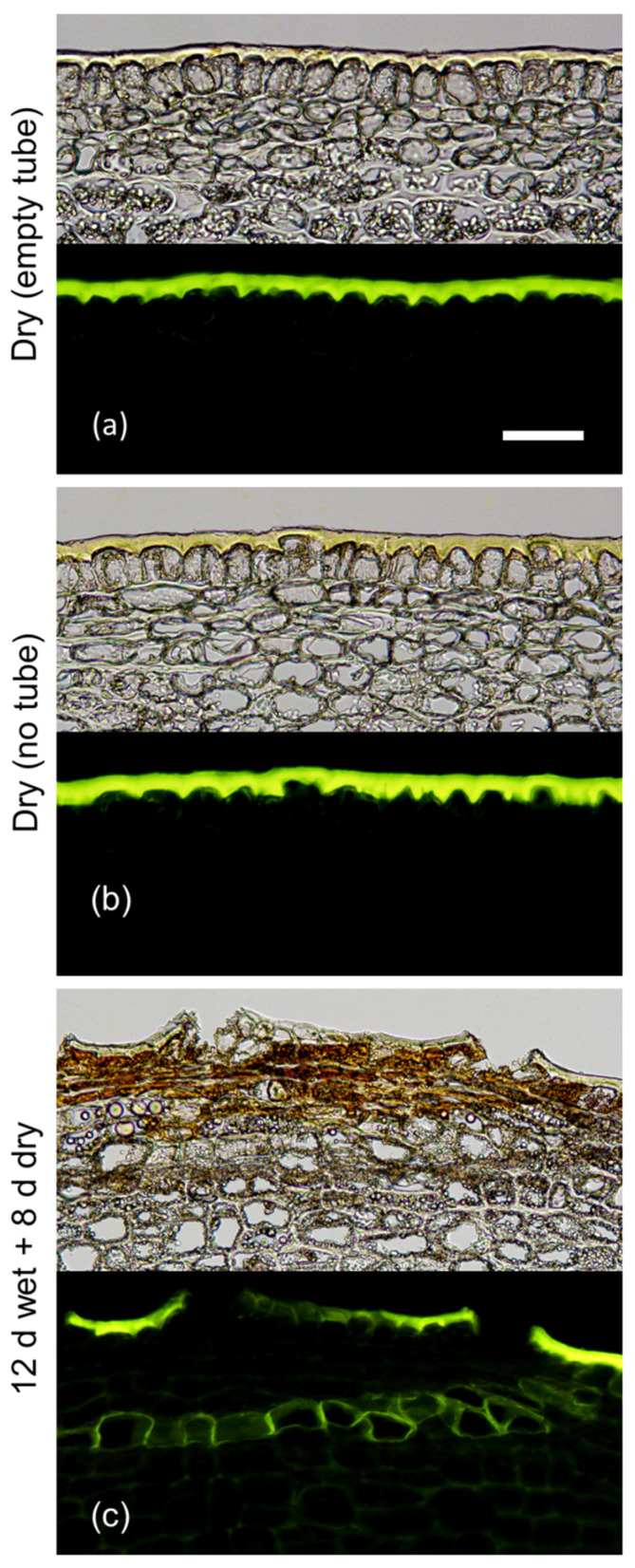
Effects of mounting tubes on the fruit surface without and with added moisture for 12 d, on the formation of periderm 8 d after removal of the tubes. (**a**) control that had a tube without water mounted for 12 d. (**b**) control without tube. (**c**) moisture treatment that had a tube containing water mounted for 12 d. The experiment comprised two phases: Phase I consisted of mounting the tube without or with water and Phase II marks the period after termination of moisture treatment. Micrographs taken under transmitted white light (upper) or incident fluorescent light (lower) (filter module U-MWB) following staining with Fluorol Yellow 088. The scale bar in (**a**) is 50 µm long and representative of all images in the composite (*n* = 3).

Following a 12 d exposure to moisture, a periderm had developed after an additional 8 d without moisture as indexed by stacks of fluorescing phellem cells visible in cross-sections of the skin (Figure 1). Furthermore, the typical russeting symptoms were visible at the fruit surface. There was no periderm and no russet visible in either of the moisture controls, regardless of the presence (or not) of the tube. Hence, we conclude that the periderm resulted from moisture exposure and not from the mounting of the tube. Because of this finding, there was no need to mount an empty tube as a control in subsequent experiments.

**Figure 2:**
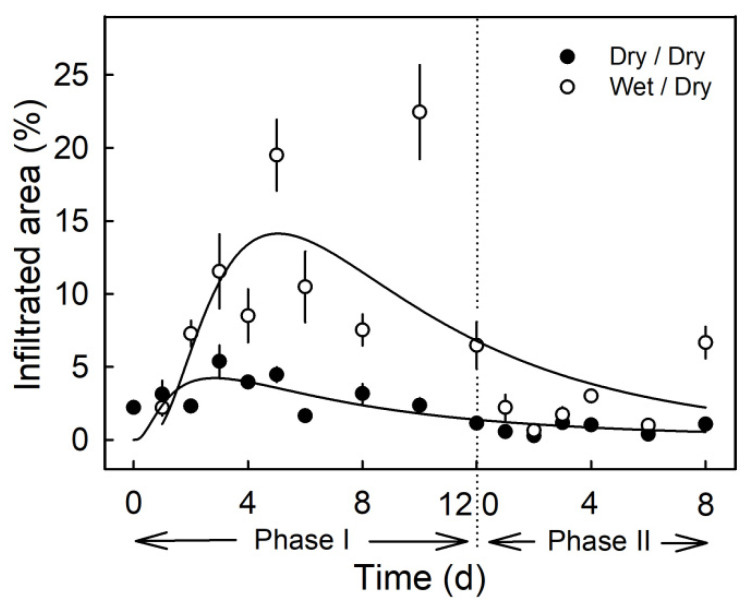
Time course of moisture-induced microcracking. Microcracking of the cuticle was indexed by quantifying the percentage of treated area infiltrated with acridine orange. The experiment comprised two phases: The first period of moisture exposure (Phase I) and the second period after termination of moisture exposure (Phase II). The end of Phase I and the beginning of Phase II is indicated by the dashed vertical line. The moisture treatment is referred to as ‘wet/dry’ and the control as ‘dry/dry.’ Data symbols present means ± SE (*n* = 6 to 20).

Moisture exposure of the fruit surface at the young stage induced microcracks in the cuticle as indexed by increased infiltration of the fluorescent tracer acridine orange (Figure 2). Moisture exposure periods of 2 to 12 d resulted in significantly higher acridine orange infiltration as compared to the non-exposed control (Phase I, Figure 2). When the moisture exposure was terminated, the area infiltrated with acridine orange decreased to a level similar to that of the non-treated control (Phase II). The only exception was at 8 d after termination of the moisture treatment. By this time, rainfall had occurred in the orchard (Phase II, Figure 2).

**Figure 3:**
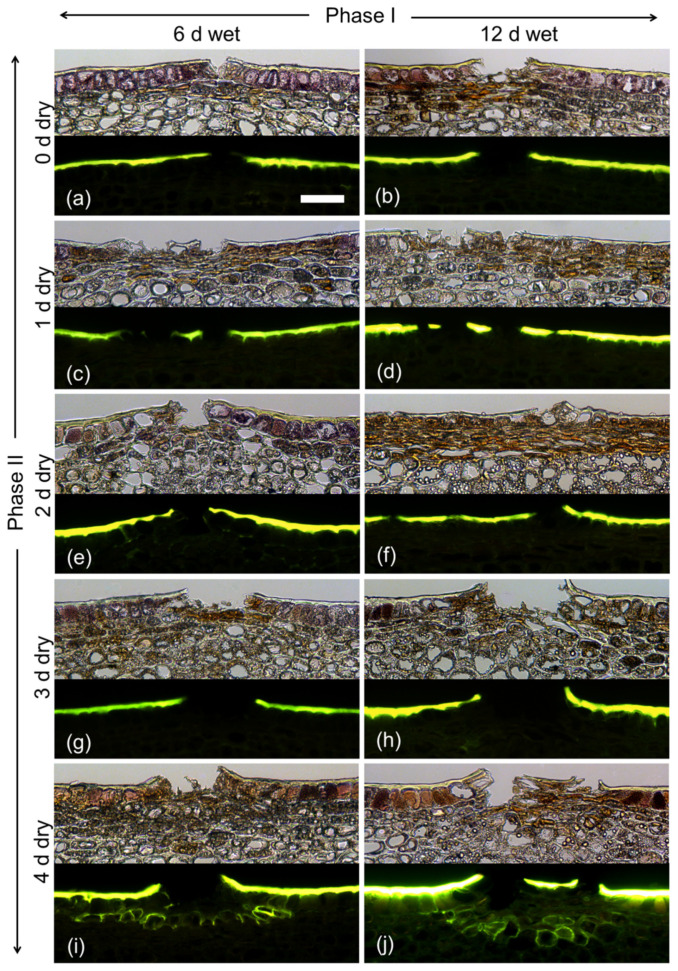
Effect of moisture exposure for 6 d (**a**,**c**,**e**,**g**,**i**) or for 12 d (**b**,**d**,**f**,**h**,**j**) on the time course of periderm development established at 0 d (**a**,**b**), 1 d (**c**,**d**), 2 d (**e**,**f**), 3 d (**g**,**h**) or 4 d (**i**,**j**) after termination of moisture exposure. The experiment comprised two phases: Phase I of moisture exposure and Phase II after termination of moisture exposure. Micrographs taken under transmitted white light (upper) or incident fluorescent light (lower) (filter module U-MWB) following staining with Fluorol Yellow 088. The scale bar in (**a**) is 50 µm long and representative of all images in the composite (*n* = 3).

During exposure to moisture (Phase I), there was no indication of periderm formation from microscopy of cross-sections stained with Fluorol Yellow 088, regardless of exposure duration (6 or 12 d; Figure 3). Microcracks had formed that traversed the cuticle. Following termination of moisture exposure (Phase II), a periderm developed by 4 d below the epidermis in the hypodermal cell layers. Periderm formation was indexed by stacks of cells that stained with Fluorol Yellow 088. These cells represented the typical cork cells (phellem) that originate from an underlying phellogen. There was no apparent difference between the periderms that formed after a 6 d or a 12 d period of moisture exposure.

**Figure 4:**
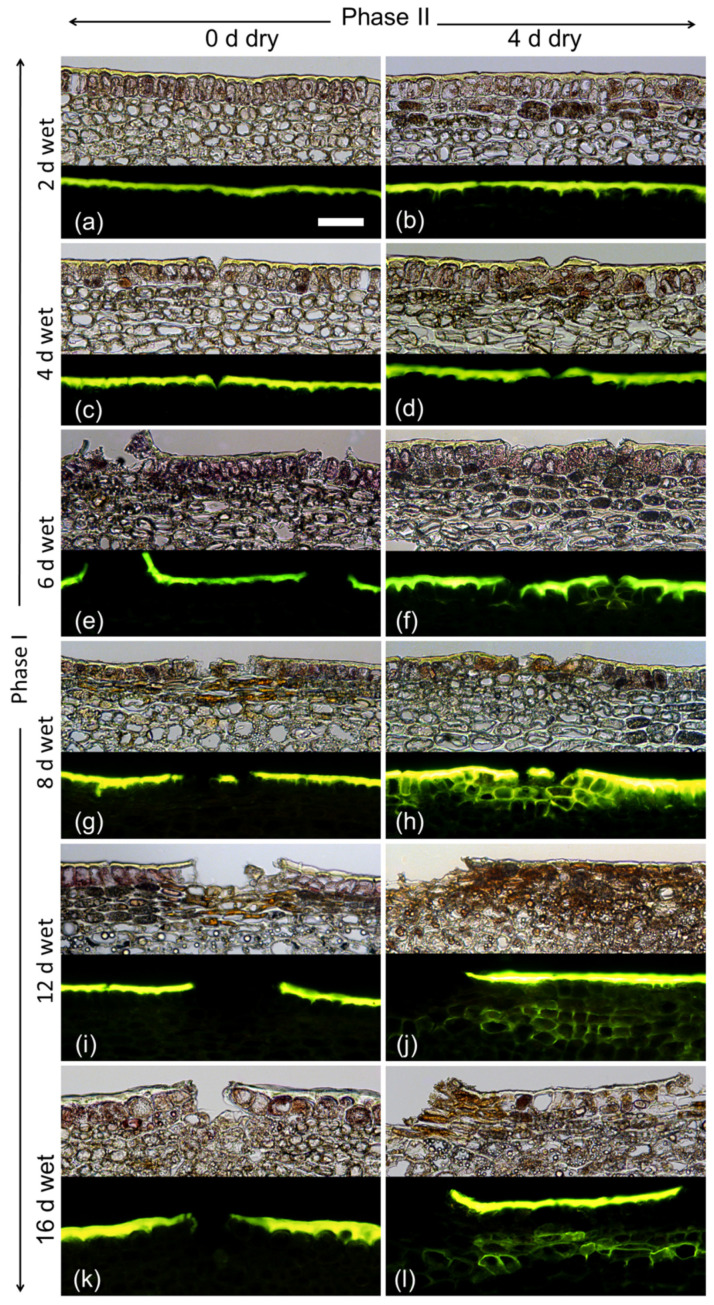
Effect of moisture exposure for 2 d (**a**,**b**), 4 d (**c**,**d**), 6 d (**e**,**f**), 8 d (**g**,**h**), 12 d (**i**,**j**) or 16 d (**k**,**l**) on periderm formation. The experiment comprised two phases: Phase I—time of moisture exposure and Phase II—time after termination of moisture exposure. Phase I was recorded immediately after termination of moisture exposure (0 d) (**a**,**c**,**e**,**g**,**i**,**k**). Phase II was recorded 4 d after termination of moisture exposure (**b**,**d**,**f**,**h**,**j**,**l**). Micrographs taken under transmitted white light (upper) or incident fluorescent light (lower) (filter module U-MWB) after being stained with Fluorol Yellow 088. The scale bar in (**a**) is 50 μm long and representative of all images in the composite (*n* = 3).

Varying the duration of moisture exposure (Phase I) revealed that a minimum moisture period of 6 d was needed to induce a periderm within 4 d after moisture termination (Phase II). As in the previous experiment, there were no detectable changes in the fruit skin during moisture exposure except for the formation of microcracks. These were observed after 4 d of moisture exposure (Figure 4).

**Figure 5:**
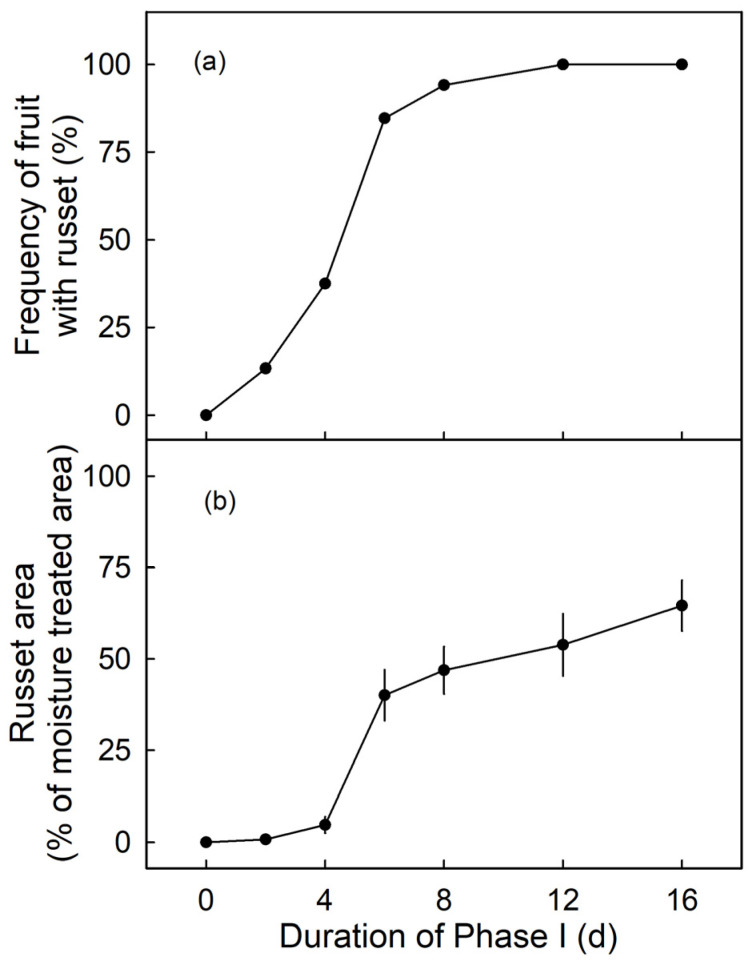
Effect of duration of moisture exposure (Phase I) on the frequency of russeted fruit (**a**) and the percentage of the moisture-exposed area that is russeted at maturity (156 days after full bloom; DAFB) (**b**). Fruits were exposed to moisture starting from 31 DAFB for 0, 2, 4, 6, 8, 12 or 16 d. Data represent means ± SE (*n* = 9–31).

The frequency of russeted fruit and the percentage of russeted area were low for moisture exposures up to 4 d (Phase I) at the young stage (from 31 DAFB onwards) but increased markedly for moisture exposures of 6 d or longer. There was little difference in frequency of russeted fruit beyond 6 d moisture exposure (Figure 5a). However, the russeted areas continued to increase from 6 to 16 d of moisture exposure (Figure 5b). There was no moisture-induced russeting at maturity (156 DAFB), when surfaces were exposed to moisture for 12 d at 66 DAFB or at 93 DAFB (*n* = 10–15; data not shown).

**Figure 6:**
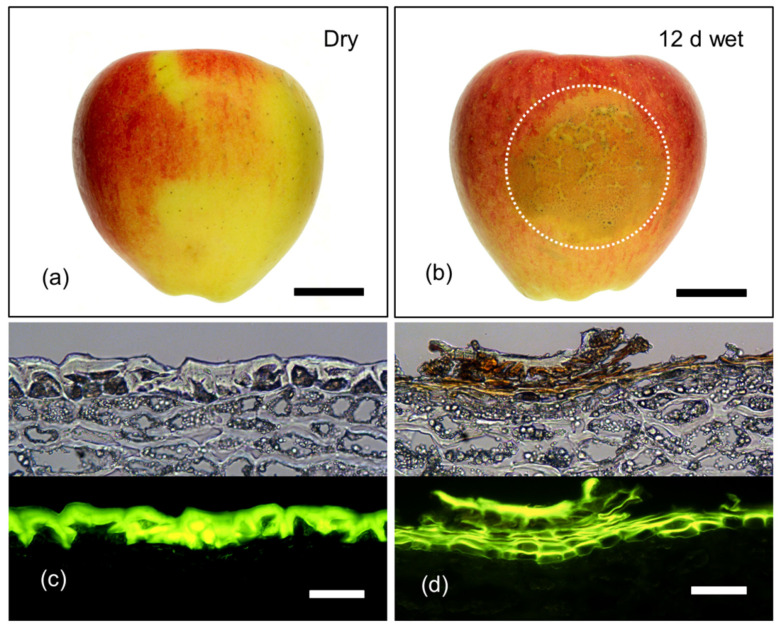
Macrographs (**a**,**b**) and micrographs (**c**,**d**) of mature (156 days after full bloom; DAFB) ‘Pinova’ apple fruit following exposure to surface moisture for 12 d at 31 DAFB (wet). Fruit without moisture-exposure, served as controls (dry). Micrographs represent cross-sections of the fruit skin in the moisture-exposed region and the dry region. Micrographs were taken under transmitted white light (upper) or incident fluorescent light (lower) (filter module U-MWB) after being stained with Fluorol Yellow 088. The area enclosed by the dotted circle represents the footprint of the moisture-treated patch of skin that subsequently developed russet. Scale bar in (**a**) and (**b**) is 2 cm long and that in (**c**) and (**d**) is 50 μm long.

Fruit exposed to moisture for 12 d beginning at 31 DAFB had developed russet at maturity (156 DAFB) and a multistack phellem typical for russeted apples was visible (Figure 6). By maturity, the cuticle and the remains of the epidermis and hypodermis had sloughed off and the brown color of the periderm was fully exposed at the surface. Furthermore, the micromorphology of the skin of moisture-treated fruit was identical to that of naturally russeted fruit of the same cultivar (data not shown).

**Figure 7:**
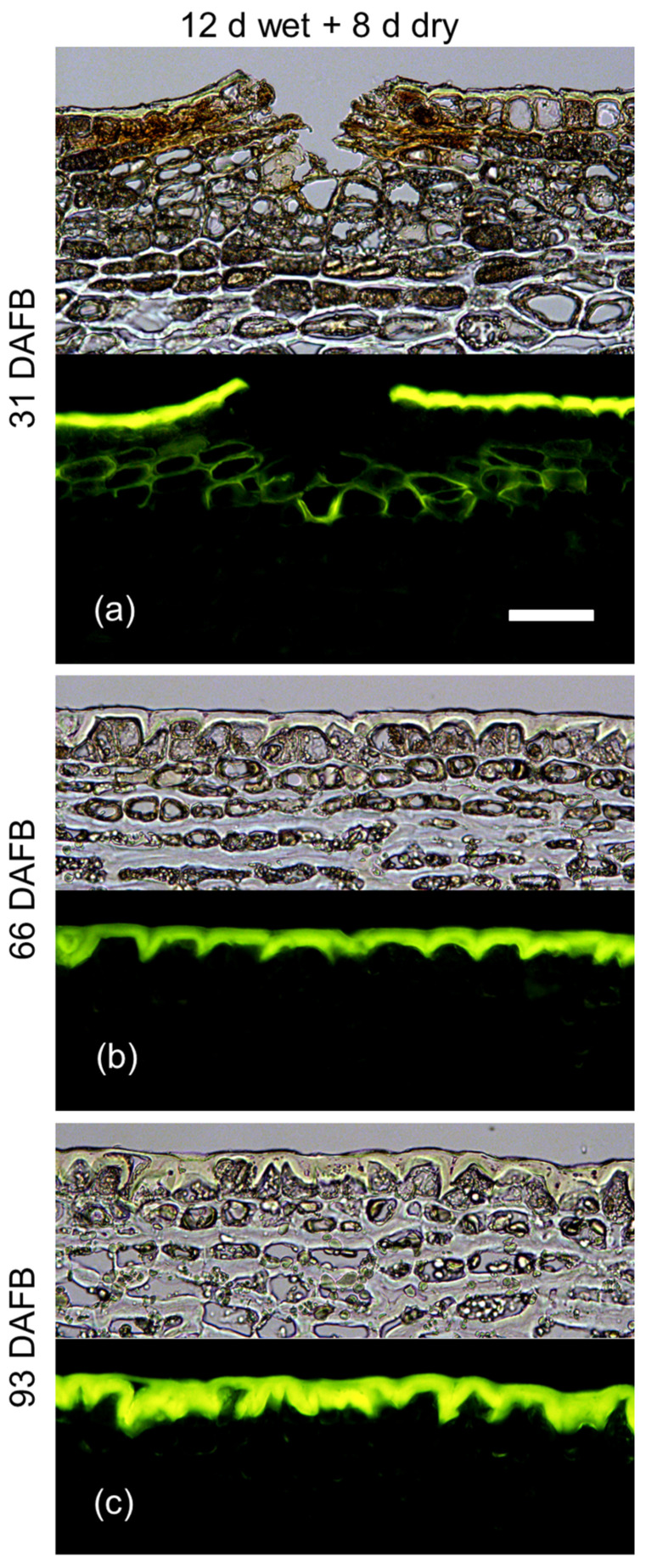
Effect of a 12 d moisture exposure (wet; Phase I) on periderm development in the skin of apple fruit. Cross-sections were prepared 8 d after termination of moisture exposure (dry; Phase II). The fruit surface was exposed to moisture starting at 31 days after full bloom (DAFB) (**a**) or 66 DAFB (**b**) or 93 DAFB (**c**). Cross-sections were prepared from the moisture-treated surface of the fruit. Images were taken under transmitted white light (upper) or incident fluorescent light (lower) (filter module U-MWB) after being stained with Fluorol Yellow 088. The scale bar in (**a**) is 50 μm long and representative of all images in the composite (*n* = 3).

The developmental time course revealed that 12 d moisture exposure induced periderm at 31 DAFB but not at 66 or 93 DAFB (Figure 7). Interestingly, microcracks were observed only following moisture exposure at 31 DAFB but not at 66 or 93 DAFB (Figure 7).

**Figure 8:**
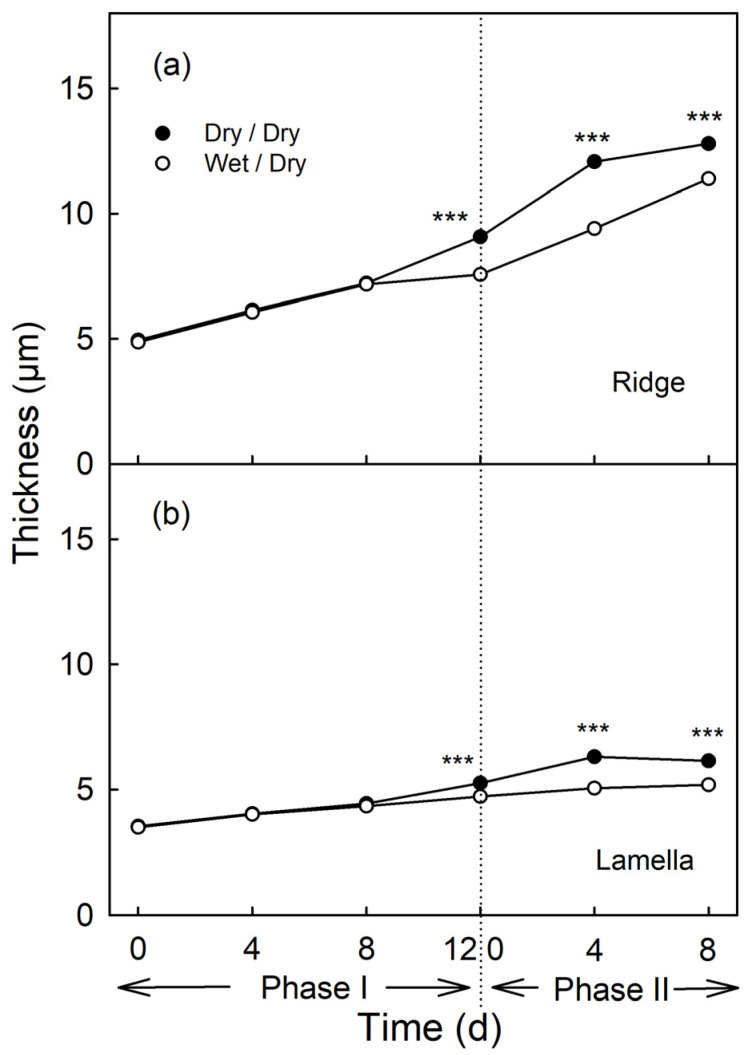
Effect of moisture exposure on the thickness of the cuticle above the anticlinal cell walls (ridge) (**a**) and above the periclinal cell walls (lamella) (**b**) of the apple fruit skin. In Phase I, the fruit was exposed to moisture for 12 d. Phase II began following termination of moisture exposure (indicated by the dotted vertical line) and the surface remained dry thereafter (wet/dry). Fruit surface without moisture exposure served as control (dry/dry). *** indicate significant difference between ‘dry/dry’ and ‘wet/dry’ treatment at *p* < 0.001. Data represent means ± SE (*n* = 6).

Moisture had no effect on cuticle thickness during the first 8 d of exposure, nor on the ridges of the cuticular membrane (CM) above the anticlinal cell walls, nor on the lamellae above the periclinal cell walls (Phase I, Figure 8). From the day of moisture removal onwards, the thickness of the cuticle of the previously exposed patch increased at a lower rate comparable to that of the non-exposed control patch (Phase II, Figure 8).

**Figure 9:**
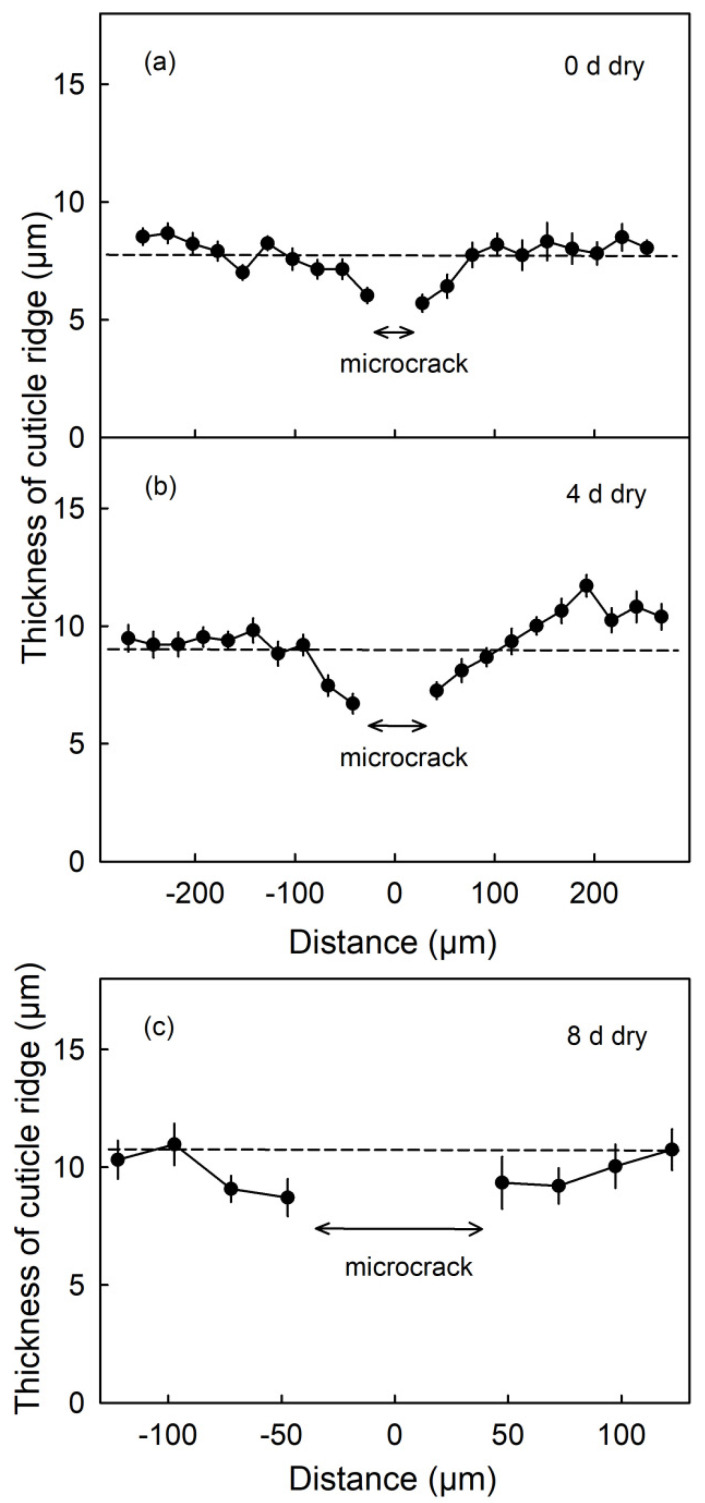
Thickness of the cuticle above the anticlinal cell walls (ridge) as affected by the distance from a moisture induced microcrack. Microcracks were induced by 12 d of moisture exposure. Thickness was measured on cross-sections of the fruit skin prepared from fruit sampled on the day of termination of moisture exposure (0 d) (**a**) and 4 d (**b**) and 8 d (**c**) after moisture termination (during Phase II). The distance ‘0’ represents the center of the microcrack. Thickness was measured in both directions from the microcrack. The dashed line is the grand mean thickness of all cuticle ridges within the micrograph. The arrows indicate the mean width of the microcrack. Data represent means ± SE of 14 to 19 microcracks on a total of six fruits.

The thicknesses of the CM ridges were lowest in the immediate vicinity of a microcrack. As distance increased, the CM thickness increased and approached the mean thickness averaged across the micrograph. This was also the case 4 d and 8 d after termination of the moisture treatment (Phase II, Figure 9).

**Figure 10:**
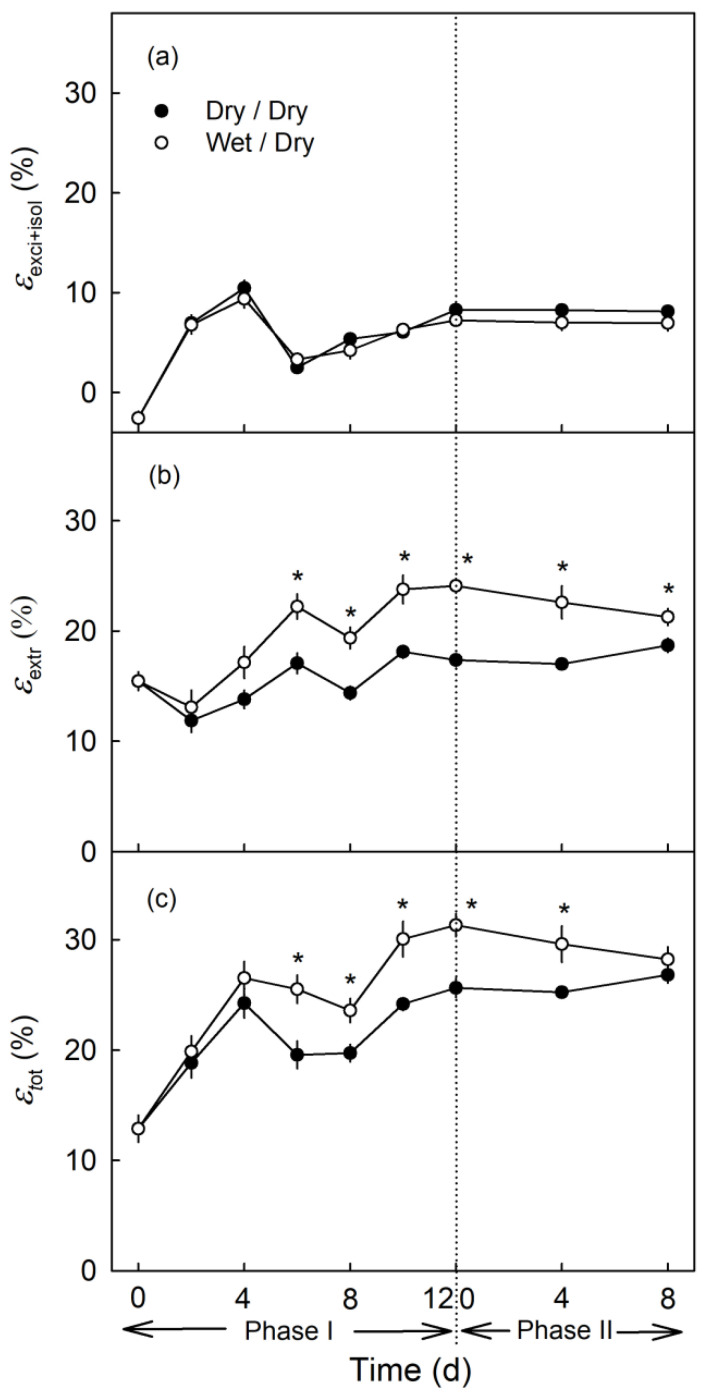
Effect of 12 d of moisture exposure (Phase I) on the elastic strain of the cuticular membrane (CM). Strain was quantified as the strain release during excision and isolation of the CM ($\varepsilon_{exci+isol}$; **a**) and following wax extraction of the CM ($\varepsilon_{extr}$; **b**) and the sum of $\varepsilon_{exci+isol}$ plus $\varepsilon_{extr}$ ($\varepsilon_{tot}$; **c**). Phase I represents the period of moisture exposure (wet). Phase II represents the period after moisture termination (dry). The dotted line indicates the end of Phase I and the beginning of Phase II. * indicates a significant difference between dry/dry and wet/dry treatment at *p* < 0.05. Data represent means ± SE (*n* = 8 to 20).

Neither moisture exposure (Phase I) and nor the termination of moisture exposure (Phase II) had an effect on strain release following preparation of the excised skin segments (ES) and isolation of the CM (Figure 10a). However, the strain release after wax extraction was higher during Phase I and after exposure to moisture (Phase II) than of the non-exposed control (Figure 10b). The difference in strain release between exposed and non-exposed CM increased up to about 6 d after the beginning of exposure and then remained approximately constant (Figure 10b). Calculating total strain from the two component strains revealed that the $\varepsilon_{tot}$ increased during moisture exposure (Phase I). The rate of increase was somewhat higher for the $\varepsilon_{tot}$ from the moisture treatment than for the control. The difference in $\varepsilon_{tot}$ decreased slightly when moisture exposure was terminated (Phase II; Figure 10c).

## 3. Discussion

Our results establish two important findings—(1) Periderm formation in young ‘Pinova’ apple fruit is not induced during moisture exposure but after termination of moisture exposure and (2) decreased rate of cuticle deposition contributes to moisture-induced microcracking. 

### 3.1. Periderm Formation in Young Fruit Is not Induced During Moisture Exposure but After Termination of Moisture Exposure

Our study is consistent with earlier observations [20]. First, microcracks traversing the cuticle are the first visible symptom in moisture-induced russeting. We have not found a single instance where russet formation was not preceded by microcracking. Second, the periderm formed in the hypodermis, beneath the cuticle and epidermis was as described by Meyer [22] and Pratt [25]. Third, early stages of fruit development were most susceptible to russet [1,20,26,27]. Indeed, no russeting occurred following exposure to moisture at later stages of fruit development. Fourth, our experimental approach provides conclusive evidence that surface moisture is the cause of russeting. A role of surface moisture in russeting has been suggested previously [18,19,24,28].

Our results consistently show that periderm formation is triggered following termination of the moisture treatment—not during it. This conclusion is based on the observation that increased durations of exposure to moisture beyond a minimum of 4 d had no effect on periderm formation. Regardless of the duration of moisture exposure, a periderm always formed about 4 d after moisture termination. This implies (1) that it is not microcracking per se that triggers russet formation and (2) that some sort of signal must be involved that has its source at the site of microcracking (the cuticle) and travels through two or three cell layers to the subtending hypodermis where the periderm is initiated. Whatever the nature of this signal, it triggers the process involved in the formation of a periderm. This process involves the dedifferentiation of a layer of cells in the hypodermis and their subsequent differentiation into a phellogen which divides repeatedly to produce a stack of suberized phellem cells [7].

Candidates for this signal could include mechanical stimuli, such as the one associated with the release of reversible strain (i.e., elastic and viscoelastic strains) when a microcrack forms in the cuticle. However, several arguments suggest this is unlikely to be the stimulus. First, there was little strain release on excision of an ES and on the isolation of the CM, thus indicating the absence of significant elastic strain in the apple fruit cuticle. This observation is consistent with an earlier one of Lai et al. [29]. Second, the contribution of the cuticle to the overall mechanical properties of the skin is small [30]. It is the epidermis and the hypodermis that together represent the structural backbone of the skin of an apple fruit. Third, if strain relaxation were a factor, one would expect periderm formation to begin after microcrack formation, that is, during moisture exposure (Phase I), not after a fixed time following termination of moisture exposure. We conclude that a mechanical signal is unlikely to be the cause.

An alternative signal candidate may be the change in the barrier properties of the microcracked cuticle. This type of signal could account for a response induced after removal of the tube. Furthermore, the remote response would also be accounted for. Changes in the chemical potential of substances for which the cuticle forms a primary barrier are probably candidates for such a signal. Following the formation of a microcrack, these substances will now move more freely across the skin. Such substances include the chemical potential of both liquid and vapor-phase water (the water potential) and the chemical potentials (partial pressures, concentrations) of dissolved moieties such as O_2_, CO_2_ and C_2_H_4_. The consequences of a suddenly less-restricted movement of water would be a change in water potential and thus of turgor. For a change of the chemical potential of the respiratory gases, for example, a decrease in [CO_2_] or an increase in [O_2_], there would likely be a change in pH. Whether these are the changes that trigger periderm formation is not known.

### 3.2. Moisture Exposure Increases Microcracking by Decreasing Cuticle Deposition

A causal role for moisture in microcracking has been documented for a number of fruit crop species including sweet cherry [31], apple [18,24], grapes [32], mango [33]. Several factors are involved in formation of microcracks. First, a mismatch of surface expansion rate and cuticle deposition rate causes increased elastic strain [29,34] leading to failure of the cuticle [35]. Second, moisture may exacerbate microcracking by altering the mechanical properties of the cuticle [23,31]. Third, our results suggest that cuticle deposition is reduced as a consequence of moisture exposure and this will likely increase microcracking. The CMs isolated from moisture-exposed regions showed a higher elastic strain than CMs from the control surfaces that remained dry. This could well have been due to decreased deposition of cuticle (cutin and wax) due to moisture exposure. That wax plays an important role, is inferred from the marked differences in strain release on extraction between the moisture treatment and the control. Earlier studies established that depositions of wax in the expanding cutin network on a growing fruit surface substantially reduce build-up of elastic strain by converting the elastic strain into a plastic strain [36]. Further, deposition of new layers of cutin underneath the existing old layers fixes the elastic strain of the CM [37]. Continuing cutin and wax deposition will therefore fix the elastic strain in the dry control skins but to a lesser extent in the skins exposed to moisture. This would result in greater strain release upon wax extraction in the control, as compared to the conditions found in the moisture treatment. Further molecular and biochemical evidence is needed to draw a firmer conclusion on this point.

### 3.3. Conclusions

The exposure of discrete patches of the fruit skin of an apple to moisture induces the formation of a periderm after termination of the moisture treatment and after the formation of microcracks. The search for a signal that links the formation of cuticular microcracks, on the fruit surface, to the initiation of dedifferentiation and redifferentiation in the hypodermis, several cell layers below, must focus on this time slot. Our results provide indirect evidence that reduced cuticle deposition and, in particular, reduced wax deposition, is the result of moisture exposure and contributes to the formation of microcracks.

## 4. Materials and Methods

### 4.1. Plant Materials

‘Pinova’ apple trees (*Malus* × *domestica*, Borkh.) grafted on M9 rootstocks were cultivated at the Horticultural Research Station of the Leibniz University Hanover at Ruthe, Germany (52°14′ N, 9°49′ E) according to current regulations for integrated crop production. The planting year was 1999, the experiments were conducted in the 2016, 2018 and 2019 growing seasons. Mean daily temperatures, mean daily precipitation and the daily radiation are provided as a supplemental file (Table S1). ‘Pinova’ was selected because it responded consistently to moisture exposure by russeting (Khanal, unpublished data). Vigorous flower clusters were selected randomly from a total of 125 trees at full bloom (0 days after full bloom; DAFB) and thinned to one flower, so that only the king flower remained. Fruitlets without visual defects and of uniform size and color were selected for the experiments.

### 4.2. General Experimental Procedures

#### 4.2.1. Moisture Treatment

Moisture was applied locally to a defined patch on the fruit surface [24]. Briefly, a polyethylene tube (8 mm inside diameter; Sarstedt, Nümbrecht, Germany) was cut to a 17 mm length and mounted on the fruit surface in the equatorial region using a non-phytotoxic, fast-curing silicone rubber (Dowsil™ SE 9186 Clear Sealant, Dow Toray, Tokyo, Japan). Deionized water was introduced through the open end of the tube and this open end was then sealed with silicone rubber. In this way, the patch of skin exposed to liquid water was limited to that enclosed within the tube (ca. 50 mm^2^). To avoid leakage, the silicone seal between tube and fruit was renewed every 2 d until the moisture treatment was terminated. An equivalent patch of skin was identified on the opposite face of the same fruit to serve as the control. Unless specified otherwise, no tube was mounted over the control patch. Earlier experiments established that russeting was due to moisture exposure and not to the mounting of the tube [24]. On the day moisture exposure was terminated, the tube was removed and the fruit surface dried with a soft paper tissue. The tube detached very easily from the epidermis, so that no significant physical force was needed and the fruit surface displayed no visible sign of injury. The footprints of the treated and control patches on each fruit were delineated using a permanent marker. A particular fruit was either sampled immediately or left on the tree for later evaluation. Following sampling, a fruit was transferred to the laboratory within 3 h. Intact fruit (21 or 31 DAFB) or sections of the fruit (66 or 93 DAFB) were stored in Karnovsky fixative [38] or immediately processed fresh, as described below.

#### 4.2.2. Microcracks

Microcracks were quantified in both the 2018 and 2019 growing seasons following the procedure described earlier [24,35]. Briefly, whole fruit were dipped in a 0.1% (*w*/*v*) aqueous acridine orange solution (Carl Roth, Karlsruhe, Germany) for 10 min, rinsed with distilled water and carefully blotted dry using a soft paper tissue. The treated and the control patches of the skin were inspected using fluorescence microscopy (MZ10F; GFP-plus filter, 440–480 nm excitation, ≥510 nm emission wavelength; Leica Microsystems, Wetzlar, Germany) and imaged with a DP71 camera (Olympus Europa, Hamburg, Germany). Three or four images were recorded from different locations within each treated or control patch, on each of a total of six to ten fruit per sampling date. The areas (mm^2^) infiltrated by acridine orange were quantified using image analysis (Cell^P^, Olympus, Hamburg, Germany). The total fluorescing area within each treated (or control) patch, in each image, was calculated and was expressed as a percentage of the whole treated (or control) patch to which it referred.

#### 4.2.3. Cross-Section of Fruit Skin

Tissue blocks (ca. 3 mm thick) comprising the fruit skin and some subtending parenchyma cells were excised from the treated or the control patches of the fixed fruit using a scalpel. The blocks were rinsed in distilled water and immersed in 70% (*v*/*v*) aqueous ethanol for 16 h. The blocks were then dehydrated in an ascending series of ethanol (80%, 90% and 96% *v*/*v*; 30 min each) under a partial vacuum (pressure 10.8 kPa). Subsequently, the blocks were transferred to 100% isopropanol for 40 min (twice) and a xylene substitute (AppliClear; AppliChem, Münster, Germany) for 40 min (twice) to displace the ethanol in the tissues, under the same partial vacuum. The dehydrated blocks were then infiltrated with a 1:1 (*v*/*v*) paraffin/xylene substitute mixture (Carl Roth) for 40 min (once) and paraffin alone for 40 min (twice). Finally, the blocks were embedded in paraffin. The paraffin blocks so obtained were cooled and stored at 4 °C pending later sectioning.

Thin sections (10 µm) were cut using a rotary microtome (Hyrax M 55, Zeiss, Germany). Sections were transferred to microscope slides, dried in an oven for 16 h at 38 °C and rehydrated as follows: xylene substitute (2 × 10 min); descending series of ethanol (96%, 80%, 70% and 60% for 10 min each) and finally for 2 × 5 min in distilled water.

#### 4.2.4. Microscopy

Sections were stained for 1 h with 0.005% Fluorol Yellow 088 (Santa Cruz Biotechnology, Texas, USA) [39] dissolved in 90% glycerol and melted polyethylene glycol 4000 (SERVA Electrophoresis, Heidelberg, Germany). The sections were transferred to the stage of a fluorescence microscope (BX-60 equipped with a DP 73 digital camera; Olympus and viewed in transmitted white light or under incident fluorescent light (filter U-MWB; 450–480 nm excitation; ≥520 nm emission wavelength; Olympus, Hamburg, Germany). The minimum number of biological replicates was three. To confirm the occurrence of a periderm, a minimum of 50 sections through the whole block were examined.

#### 4.2.5. Cuticle Thickness Measurement

Cross-sections of the skin from the moisture treated and the control patches were inspected at ×200 in white light using a fluorescence microscope (BX-60; Olympus, Hamburg, Germany). The thickness of the CM above the anticlinal cell walls (ridge) or above the periclinal cell walls (lamella) were measured in two sets of images using image analysis (CellSens; Olympus, Hamburg, Germany). The first set comprised images selected for the absence of cuticular cracks. The thickness of the lamella and ridge were measured in a 350 µm long transect. For this, four images per fruit from a total of six fruits were used. For the second set, images were selected which had a single cuticular crack. Here, the width of the crack and the thickness of the cuticular ridges were measured in a 275 µm (0 d and 4 d) or 125 µm (8 d) long transect from the center of the crack to either side. A total of 14 to 19 images on six fruits were used.

#### 4.2.6. Russet Quantification

Mature fruit were harvested at 156 DAFB. Digital calibrated images (Canon EOS 550D, lens: EF-S 18-55 mm, Canon Germany, Krefeld, Germany) were taken from the moisture treated and control patches on the fruit surface. The areas (mm^2^) of the russeted spots on the fruit surface (as indexed by their brownish, rough, corky appearance) were quantified (Cell^P^; Olympus, Hamburg, Germany) and summed within each patch of skin enclosed by the tube. The area of russet is expressed as a percentage of the area of the patch. The number of replicates ranged from 9 to 31.

#### 4.2.7. Cuticle Isolation and Strain Analysis

The ES were punched from the treated and control patches using a biopsy punch (8 mm diameter; Kai Europe, Solingen, Germany; 10 and 12 mm diameter; Acuderm, Terrace, FL, USA). The CMs were isolated enzymatically by incubating the ES in an isolation medium containing pectinase (9%, *v*/*v*; Panzym Super E flüssig; Novozymes A/S, Krogshoejvej, Bagsvaerd, Denmark) and cellulase (0.5% *v*/*v*; Cellubrix L.; Novozymes A/S) in a 50 mM citric acid buffer at pH 4.0 at ambient temperature [40]. NaN_3_ was added at a final concentration of 30 mM to prevent microbial growth. Enzyme solutions were replaced periodically until CM separated from adhering cellular debris (about 4 weeks). The isolated CMs were carefully cleaned using a soft camel-hair brush. The CM were rinsed in distilled water, dried at 40 °C for a minimum period of 16 h and stored in multi-well cell culture plates held in polyethylene boxes above dry silica gel. For determination of the wax mass, the CM discs were extracted for 2 h using CHCl_3_/MeOH (1:1, *v*/*v*; Carl Roth) in a Soxhlet apparatus. The dewaxed CMs are referred to as DCMs.

The elastic strain was quantified using the procedure described in Lai et al. [29] with minor modifications. The CMs were rehydrated, placed on a microscope slide, flattened by placing a coverslip on top and then imaged under a dissecting microscope (Wild M10; Leica Microsystems; camera DP71). For the DCMs, the discs were transferred from the CHCl_3_/MeOH to MeOH and then directly to water, before being positioned on a microscope slide and flattened as described above. The areas of the CM and DCM discs were quantified by image analysis (Cell^P^; Olympus, Hamburg, Germany).

The strains released following excision of the ES and isolation of the CM ($\varepsilon_{exci+isol}$) and following wax extraction ($\varepsilon_{extr}$) were calculated as follows:

(1) $$\varepsilon_{exci+isol}=\frac{A-A_{CM}}{A_{DCM}}\times 100$$

(2) $$\varepsilon_{extr}=\frac{A_{CM}-A_{DCM}}{A_{DCM}}\times 100$$

(3) $$\varepsilon_{tot}=\varepsilon_{exci+isol}+\varepsilon_{extr}.$$

In this equation, A represents the area of the disc on the fruit surface before excision, that is, the cross-sectional area of the biopsy punch corrected for curvature of the disc. The $A_{CM}$ and $A_{DCM}$ represent the areas of the isolated CM and the extracted DCM. Because the $\varepsilon_{exci+isol}$ and the $\varepsilon_{extr}$ are additive, the total strain $\varepsilon_{tot}$ equals the sum of the two component strains. The number of replicates ranged from 8 to 20.

### 4.3. Experiments

All experiments were conducted in two phases: the moisture treatment was imposed during Phase I. The moisture treatment was then terminated, the tube removed and the treated patch now opened up to the natural atmosphere of the orchard—this second period was Phase II. The following experiments were conducted:

(1) The first experiment established that moisture exposure was the cause of periderm formation (and not the mounting of a polyethylene tube using silicone sealant). The experiment was conducted at 28 DAFB and comprised a control (without tube, without water) and the following two treatments: (i) an empty 8.5 mm long tube (no added water) with its distal end left open to the atmosphere and (ii) a moisture treatment in which an attached 17 mm long tube was filled with water and its distal end sealed with silicone sealant. The tube in (i) was half length so as to minimise any increase in humidity in the tube—earlier experiments showed that microcracking can also result from exposure to high humidity [16,19]. This tube was also mounted in such a position that, although open to the atmosphere, rainwater could not enter it. All tubes were removed after 12 d and the fruit sampled for histological analysis after a further period of 8 d in the orchard.

(2) The time course of the duration of exposure to the atmosphere (Phase II) following removal of surface moisture was studied. The fruit surface was exposed to moisture at 31 DAFB (2019 season) for 6 or 12 d when the moisture treatment was terminated and the time course of exposure to the atmosphere began. Fruit were sampled for microcracking, CM strain and histology at 0, 1, 2, 3 or 4 d after termination of moisture exposure (Phase II) or at maturity (156 DAFB).

(3) The time course of the duration of moisture exposure (Phase I) was studied by exposing fruit surfaces from 21 DAFB (2018 season) or 31 DAFB (2019 season) onwards to moisture for 0, 2, 4, 6, 8, 12 or 16 d. Fruit were sampled either immediately after termination of the moisture treatment for microcracking, CM strain and histology or at maturity (156 DAFB) to quantify the frequency of fruit with russet and the percentage of russeted surface area.

(4) A developmental time course was established to identify any changes in periderm formation during fruit development. Moisture was applied to the surface of developing fruit, beginning at 31, 66 or 93 DAFB (2019 season) for 12 d (Phase I) and fruit were sampled 8 d after termination of the moisture treatment (Phase II). At this time, any periderm formed was clearly detectable by microscopy. Some fruit were left on the tree, sampled at maturity (156 DAFB) and used to quantify the frequency of fruit with russet and the percentage of russeted surface area.

### 4.4. Data Analyses and Presentation

Data are presented as means ± SE. Where error bars are not visible, they were smaller than data symbols. Data for strain relaxation analysis and cuticle thickness were subjected to one-way analysis of variance (ANOVA) using SAS (Version 9.1.3; SAS Institute, Cary, NC, USA). Means were compared using Tukey’s studentized test at *p* ≤ 0.05.

## Supplementary Materials

The following is available online at https://www.mdpi.com/2223-7747/9/10/1293/s1, Supplementary Table S1: Meteorological data.
